# The effect of etanercept therapy on adrenal steroid metabolism in juvenile idiopathic arthritis: a steroid metabolomics approach

**DOI:** 10.1186/s12969-023-00813-y

**Published:** 2023-04-12

**Authors:** Yonatan Butbul Aviel, Ariel Keinan, Michaela F. Hartmann, Stefan A. Wudy, Dov Tiosano

**Affiliations:** 1 Department of Pediatrics B, Ruth Rappaport Children’s Hospital of Haifa, Rambam Medical Center, 1 Efron Street, Bat-Galim, Haifa, 31096 Israel; 2 Pediatric Rheumatology Service, Ruth Rappaport Children’s Hospital, Rambam Medical Center, Haifa, Israel; 3 Bruce Rappaport Faculty of Medicine, The Technion, Haifa, Israel; 4 Pediatric Endocrinology & Diabetology, Laboratory for Translational Hormone Analytics in Pediatric Endocrinology, Steroid Research and Mass Spectrometry Unit, Center of Child and Adolescent Medicine, Justus Liebig University, Giessen, Germany; 5 Division of Pediatric Endocrinology, Ruth Rappaport Children’s Hospital, Rambam Health Care Campus, Haifa, Israel

**Keywords:** Juvenile idiopathic arthritis, Steroid, Metabolomics, GC-MS, Anti-TNFα

## Abstract

**Objective:**

To evaluate the impact of anti-tumor necrosis factor-alpha (TNFα: etanercept [Etanercept ®]) therapy on adrenal activity in juvenile idiopathic arthritis (JIA) .

**Method:**

Eleven JIA patients aged 12 ± 6.2 years with a disease duration of 6.3 ± 5.2 years were enrolled. They were treated once weekly with etanercept (0.8 mg/kg) for 3 ± 2.8 years. Urine samples for gas chromatography-mass spectrometry steroid hormone analysis were collected before, and 1 and 3 days after etanercept injection and compared to age- and sex-matched healthy controls.

**Results:**

The levels of 21 of the 31 metabolites were low before etanercept treatment. Those 21 metabolites included 4 C19 steroids (androgens), 5 C C21 steroid hormone intermediates, 10 cortisol metabolites, and 2 corticosterone metabolites. One day after treatment, only 5 of the 21 metabolite levels remained low. They included 2 C19 metabolites, 2 C21 steroid metabolites and 1 cortisol metabolite β –Cortol (β-Cl). Three days after treatment, the only metabolites levels that continued to be low were 2 C19 metabolite, 2 C21 steroid hormone intermediates and 1 cortisol metabolite α-Cortol (a-Cl), while the remaining 15 metabolites had already normalized after 1 day. Dehydroepiandrosterone-sulfate and 17-hydroxypregnenolone metabolite levels were the last ones to recover. Urinary metabolite ratios reflecting cytochrome P450 CYP21A2 (21-hydroxylase) and 11β-hydroxysteroid dehydrogenase type 2 (11β-HSD2) enzymatic activitieswere lower in JIA patients than in controls, although significant was not reached.

**Conclusion:**

Almost all of the pre-etanercept treatment cortisol urinary metabolite levels were significantly lower than normal, and almost all rose to normal values by 1 day after treatment. The therapeutic effect of anti-TNFα treatment in JIA may be related to its effect on the restoration of adrenal function and cortisol levels.

## Key message

**What is already known about this subject?**

• Serum cortisol concentrations are disproportionally low relative to the level of inflammation in rheumatoid arthritis (RA) and other inflammatory diseases.

• Tumor necrosis factor-alpha (TNFα) is elevated in the serum and the synovial fluid in children with juvenile idiopathic arthritis (JIA).

• Anti-TNFα treatment has an effect on the hypothalamic–pituitary–adrenal axis and improves adrenal hormone secretion in adults with RA. Comparable findings were never confirmed in pediatric JIA patients.

**What does this study add?**

**How might this impact on clinical practice or future developments?**

• The current study introduces the concept that the urinary steroid metabolome is suitable for assessing and monitoring the disease activity in JIA as well as for predicting disease flare.

## Introduction

Juvenile idiopathic arthritis (JIA) is the most common rheumatic disease in children and a major cause of functional disability. The chronic inflammatory synovitis and systemic features of JIA are mediated by the cytokine products of an activated immune system. Among the many cytokines that are involved in the acute phase, the levels of interleukin-6 (IL-6), IL-8 and tumor necrosis factor alpha (TNFα) are the most predominantly elevated. TNFα is one of the proinflammatory cytokines that has a complex role in the pathogenesis of rheumatoid arthritis (RA) [1] [2] [3]. It is elevated in the serum and in the synovial fluid in children with JIA [3].

Cortisol is a major anti-inflammatory substance whose low levels during the evening and night are linked to an increase in proinflammatory cytokines, such as TNFα and IL-6, during the early morning. That rise in cortisol levels in the early morning is related to the inhibition of inflammation by cytokines during the day [4] This cortisol circadian rhythm is clinically linked to pain and stiffness, which are prominent during the early morning in JIA. Several studies have observed that serum cortisol concentrations in adults rheumatoid arthritis (RA) and other inflammatory diseases are disproportionally low relative to the level of inflammation [5] Bilginer et al. showed that JIA patients with active disease have low early morning serum cortisol levels as well as low adrenocorticotropic hormone (ACTH) levels that correlate with high IL-6 levels as seen in active disease [6]. In line with that observation, the findings that urinary free cortisol levels in active JIA patients are lower than those in healthy controls and that they rise during periods of remission in parallel to the serum cortisol levels suggest that the activity of the Hypothalamus-Pituitary-Adrenal (HPA) axis is affected during the active phase of JIA [6] [7].

The hypocortisolemia in JIA may result either from primary or secondary hypoadrenalism. The endocrine profile in some untreated RA patients revealed elevated adrenocorticotropic hormone (ACTH) levels without hypercortisolemia, a combination that reflects resistance to ACTH or the presence of primary hypoadrenalism [8] [9]. These observations correspond with a seminal study which showed that TNFα is a potent inhibitor of adrenocorticotropin-induced cortisol production and steroidogenic P450 enzyme gene expression in cultured human fetal adrenal cells. [8] Other studies in patients with JIA showed low levels of ACTH, serum cortisol and urinary free cortisol, a combination that reflects a disturbance in the hypothalamic pituitary adrenocortical axis (HPA) [5] [6] .

Etanercept (Enbrel®, Immunex, Seattle), a genetically engineered fusion protein consisting of 2 identical chains of the recombinant extracellular human TNF-receptor p75 monomer fused with the Fc domain of human immunoglobulin1, effectively binds TNFα and lymphotoxin-α and inhibits their activity. Treatment with etanercept reportedly leads to significant improvement in patients with active polyarticular JIA [10].

The aim of this study was to evaluate the role of etanercept on adrenal steroid hormone metabolism by assessing the urinary steroid metabolome in JIA.

## Methods

### Patients

The study was approved by the Rambam Helsinki Committee (RMB) and all patients signed an informed consent (0054 − 14 RMB). The study group was composed of all consecutive patients who were treated with once weekly etanercept injections and followed in the Pediatric Rheumatology Service in Rambam Hospital (Haifa, Israel). The patients were matched with a healthy control based on their age, gender and Tanner stage. The diagnosis of JIA was made according to the JIA criteria of the International League of Associations for Rheumatology classification [11]. Disease activity was evaluated by measuring the number of active joints and C-reactive protein (CRP) levels. Patients who were treated with corticosteroids by oral or intra-articular injection during the preceding 3 months were excluded.

### Methods

Urinary samples were obtained from all patients. The first sample was collected on the day before Etanercept injection. The second and the third samples were collected one day and three days after the injection. One urine sample was obtained from healthy controls. All urine samples were spot urines from first morning voiding.

The patient took urine samples at home, and the parents requested that the urine will be frozen as soon as possible. All the urine samples were than collected on the same day by our team member and transported freeze to the lab.

Urinary steroid metabolites were analyzed by using quantitative data that were generated by gas chromatography-mass spectrometry (GC-MS) analysis as described previously [12] [13]In brief, free and conjugated urinary steroids were extracted by solid phase extraction (Sep-Pak C18-cartridges, Waters) from a 5-mL urinary aliquot, and conjugates were enzymatically hydrolyzed (sulfatase from helix pomatia type H-1, Sigma-Aldrich, 37 °C, 48 h). After recovery of hydrolyzed steroids by solid phase extraction, known amounts of internal standards (312,5 ng of 5α-androstane-3α, 17α-diol and stigmasterol, respectively) were added to each extract before formation of methyloxime-trimethylsilyl ethers (2% methoxyamine hydrochloride, Sigma-Aldrich, in pyridine; trimethylsilylimidazole, Macherey-Nagel). After purification by gel chromatography (Lipidex-5000, Perkin Elmer) the derivative was dissolved in 500 µL of isooctane. Organic solvents were purchased from Merck. GC was performed using an Optima-1 fused silica column housed in an Agilent Technologies 6890 series GC that was directly interfaced to an Agilent Technologies 5975 mass selective detector. Injections (1 µL) were made into an 80 °C (2 min) oven, in which the temperature was increased by 20 °C/min to 190 °C (1 min). To separate steroids the temperature was subsequently increased by 2.5 °C/min to 272 °C. For each analyte the monitoring of two typical fragment ions (target ion and qualifier ion) ensured specificity [12]. Quantitation took place in the linear range of the calibration plots of our analytes. For all urinary steroids measured, intra assay precision varied between 1.7 and 9.5% and inter assay precision ranged between 1.1 and 9.5% [13]. Values of 31 metabolites identified by GC-MS were compared between JIA patients and healthy controls. This generated a metabolic profile of JIA patients before and after Etanercept treatment compared to healthy controls.

### Statistical analysis

The results are expressed as median and the 25 and 75 percentiles. The paired T-test was applied in for data with normal distribution and Wilcoxon test was applied to compare the data without normal distribution. Fisher’s exact test was used to compare the number of metabolites whose values were significantly different from the control values, after which Bonferroni correction for multiple comparisons was applied. Significance was determined at p values < 0.05.

## Results

Eleven JIA patients were enrolled into the study. There were 8 females and 3 males with an age range from 3 to 21 years. Five patients had polyarthritis, 3 had oligoarthritis, 2 had psoriatic arthritis, and 1 had systemic arthritis. The disease duration ranged from 2 to 18 years. The duration of etanercept treatment ranged from 6 months to 10 years. Two patients were also treated with methotrexate during the study (patients 1 and 2, Table 1).

**Table 1:** Clinical characteristic of the patients with juvenile idiopathic arthritis

	Sex	Age	JIA subtype	Disease duration (years)	No. of active joints	CRP levels * (mg/dL)	Treatment duration with etanercept (years)	Etanercept dosage (mg*1/w)
1	F	16	Psoriatic arthritis	3	0	3.6	0.7	50
2	F	16	Oligoarthritis	4	0	< 0.5	3	50
3	M	10	Polyarthritis	9	0	4.5	0.8	25
4	F	21	Oligoarthritis	18	3	n/a	10	50
5	M	8	Polyarthritis	3	1	5.5	3	25
6	F	15	Polyarthritis	7	0	8.6	6	50
7	F	18	Oligoarthritis	15	0	n/a	5	50
8	M	5	Systemic	4	0	< 0.5	3	25
9	F	15	Polyarthritis	4	0	10.1	3	50
10	F	15	Psoriatic arthritis	6	1	< 0.5	1	50
11	F	3	Polyarthritis	2	0	< 0.5	0.6	7.5

n/a, not available * normal levels 0–5 mg/dL

Thirty-one urinary metabolites that provide an integrated picture of human steroid hormone metabolism were analyzed before and after etanercept treatment (Table 2, Fig − 1). The levels of 21 of those 31 metabolites were significantly lower than normal before treatment. The 21 metabolites included 4 of the 11 C19 steroids (androgens), 5/8 C21 steroid hormone intermediates, 10/10 cortisol metabolites, 2/3 corticosterone metabolites (Table 2, Fig − 1). One day after etanercept treatment, only five out of the 21 metabolites remained significantly lower than control group. The five metabolites were 2 C19 metabolites (androsterone (An) and 5a-androstane-3α), 11β-diol-17-one (11OH-An) and 2 C21 steroids [pregnenediol (P5D preg) and 11-O-Pt (5b-Pregnane-3a,17a, 20a-triol-11-one)]. Three days after etanercept treatment, five metabolites were significantly low: 2 C19 steroid metabolites An and 11OH-An, 2 C21 intermediates Pt and P5D and 1 cortisol metabolite α-Cortolone (a-Cl) (Fig-2 and Fig − 3).

**Table 2:** Urine metabolite values in the juvenile idiopathic arthritis patients and controls

Metabolite	Before Etanercept		One day after Etanercept		Three days after Etanercept			Control group
	Median, (25%;75%)µg/L	p	Median, (25%;75%)µg/L	p	Median, (25%; 75%) µg/L		p	Median, (25%; 75%) µg/L
**C19-steroids (androgens)**								
An	386 (197.7 ; 901.9)	0.003	575.66 (143.39 ; 847.7)	0.019	412.14 (195.7 ; 847.17)		0.008	1027.3 (635.93 ::1815.4)
Et	322.81 (161.99 ; 763.55)	0.09	513.98 (100.64 ; 801.78)	0.4	394.96 (133.79 : 875.92)		0.4	1083.13 (517.60 ; 2262.37)
DHEA	23.96 ( 18.12 ; 115.51)	0.8	41.73 (23.26 ; 208.48)	0.8	22.94 (17.43 ; 178)		0.4	84.64 (32.7 ; 157.21)
16a−OH−DHEA	80.98 (16.93 ;184.35)	07	125.81 (41.37 ; 297.4)	0.9	93.25 (20.86 ; 240.19)		0.8	201.56 (.80.67 ; 378.087)
A5−3b,17a	13.88 )6. 57; 26.5)	0.0049	14.94 (0; 159.06)	0.07	19.03 (0.00; 175.92)		0.2	45.22 (11.03; 112.41)
A5−3b,17b	18.44 (0. 5; 58.45)	0.3	19.34 (0.5 ; 19.34)	0.31	20.91 (0.5 ; 44.91)		0.3	39.52 (4.24 ; 67.7)
A5T−16a	69.89 (25.18;187.62)	0.9	81.11 (25.11;183.22)	0.9	79.34 (32.86;210.62)		0.6	136.71 (102.88;443.62)
11−OH−An	258.05 (140.19;328.16)	0.0053	193.16 (122.67;476.65)	0.039	241.12 (44.47; 1281.82)		0.047	540.11 (256.35;750.63)
11−O−An	23.58 (16.15;30.91)	0.028	21.35 (18.63;40.38)	0.1	25.65 (17.6;29.15)		0.09	40.94 (31.03;68.16)
11−OH−Et	53.93 (32.22;143.80)	0.2	134.69 (24.96;159.60)	0.5	60.74 (27.94;172.20)		0.68	311.31 (192.76;555.11)
C21-steroids Pregnenolone and progesterone metabolites								
PD	87.33 (22.35;154.31)	0.4	53.86 (24.95;150.32)	0.5	55.49 (29.01;217.23)		0.3	167.63 (82.29;251.39)
PT	243.94 (129.83;456.88)	0.007	221.14 (134.19;442.83)	0.2	279.72 (144.10;521.28)		0.001	534.55 (360.57;814.75)
P5D	62.65 (26.36;79.76)	0.015	38.94 (15.78;72.36)	0.013	47.28(29.65;102.80)		0.009	143.12 (89.79;191.43)
P5T−17a	92.30 (22.90;187.19)	0.11	91.35 (45.7;222.31)	0.3	97.89 (40.90;256.69)		0.2	236.23 (108.73;375.27)
Po−5b,3a	37.16 (19.60;63.17)	0.0001	28.40 (17.13;91.86)	0.08	45.78 (24.49;119)		0.1	76.18 (62.33;144.34)
Po−5a,3a	8.6 (4.37;11.15)	0.064	7.08 (4.80;15.49)	0.1	9.70 (0.00; 29.03)		0.1	12.19 (7.73; 83.24)
11−O−Pt	5.14 (2.39; 41.76)	0.0018	3.98 (2.21; 15.49)	0.03	9.10 (4.86;13.61)		0.8	11.83 (9.05;16.24)
THS	58.26 (38.47;119.66)	0.0065	79.51 (35.70;130.07)	0.1	70.71 (51.71;125.88)		0.16	93.25 (61.12;193.81)
Cortisol metabolites								
F	39.51 (30.95;53.47)	0.025	35.64 (27.98;72.95)	0.7	52.76 (30.74;68.72)		0.5	68.94 (45.66;102.47)
THE	2030.35 (929.30;3716.08)	0.022	1881.50 (1079.42;2993.02)	0.6	2211.90 (1709.79;2867.23)		0.2	3308.17 (2853.92;4929.21
THF	846.06 (373.61;1043.06)	0.028	620.38 (414.52;885.59)	0.4	814.63 (526.79;1045.78)		0.3	1029.31 (817.97;1671.19)
a−THF	434.07 (337.48;755.61)	0.016	552.75 (354.94;621.4)	0.3	493.77 (46.30; 2137.91)		0.1	846.49 (378.17;1162.60)
a−Cl	617.24 (356.03;1043.48)	0.012	643.37 (395.74;840.70)	0.6	653.12 (504.40;954.33)		0.03	1187.74 (763.86;1677.21)
b−Cl	374.98 (278.97;773.43)	0.001	447.54 (262.38;789.72)	0.2	480.85 (411.58;586.12)		0.08	703.25 (535.32;1107.11)
a−C	74.67 (52.41;154.94)	0.002	85.60 (59.87;138.94)	0.07	106.13 (61.91;149.26)		0.08	167.22 (89.83;295.84)
b−C	223.18 (118.71;414.28)	0.014	298.90 (160.79;519.41)	0.045	329.43 (145.79;583.53)		0.07	447.79 (260.20;807.25)
6b−OH−F	85.51 (58.81;234.72)	0.02	94.19 (68.37;178.74)	0.1	126.28 (82.995;182.24)		0.5	247.6 (93.65;313.36)
20a−DHF	24.52 (11.98;29.30)	0.03	23.54 (15.96;33.74)	0.1	26.02 (.16.04;41.29)		0.3	36.31 (24.38;57.84)
Corticosterone metabolites								
THA	88.06 (68.73;174.89)	0.034	86.80 (59.50;199.31)	0.49	121.73 (81.15;153.81)		0.07	225.99 (89.56;343.93)
THB	97.58 (53.39;157.32)	0.025	75.08 (54.44;169.64)	0.3	106.76 (81.92;196.15)		0.052	223.62 (124.41;274.19)
a−THB	153.63 (109.67;271.18)	0.053	163.06 (106.41;316.88)	0.3	161.34 (123.49;292.23)		0.09	286.74 (119.17;655.65)

Significant values (p < 0.05)

P values are versus controls

**Fig. 1:**
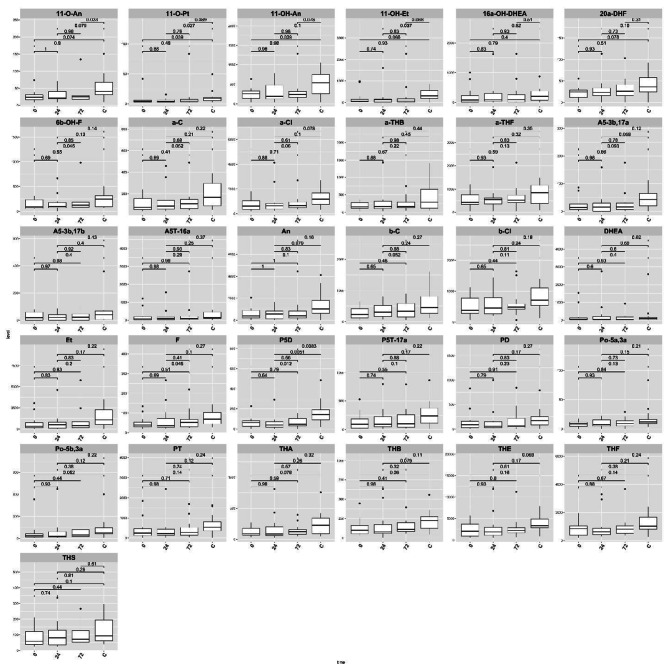
The X axis represent the time of the urine sample 0,24.72 h following the Etanercept injection The box plot graph represents the levels of the 21 metabolites in 0,24.72 h following the Etanercept injection The black dots represent the outliers of 21 metabolites in 0,24.72 h following the Etanercept injection The upper part of the graph represents differences between the metabolites levels in 0,24.72 h following the Etanercept injection and the control

**Fig. 2:**
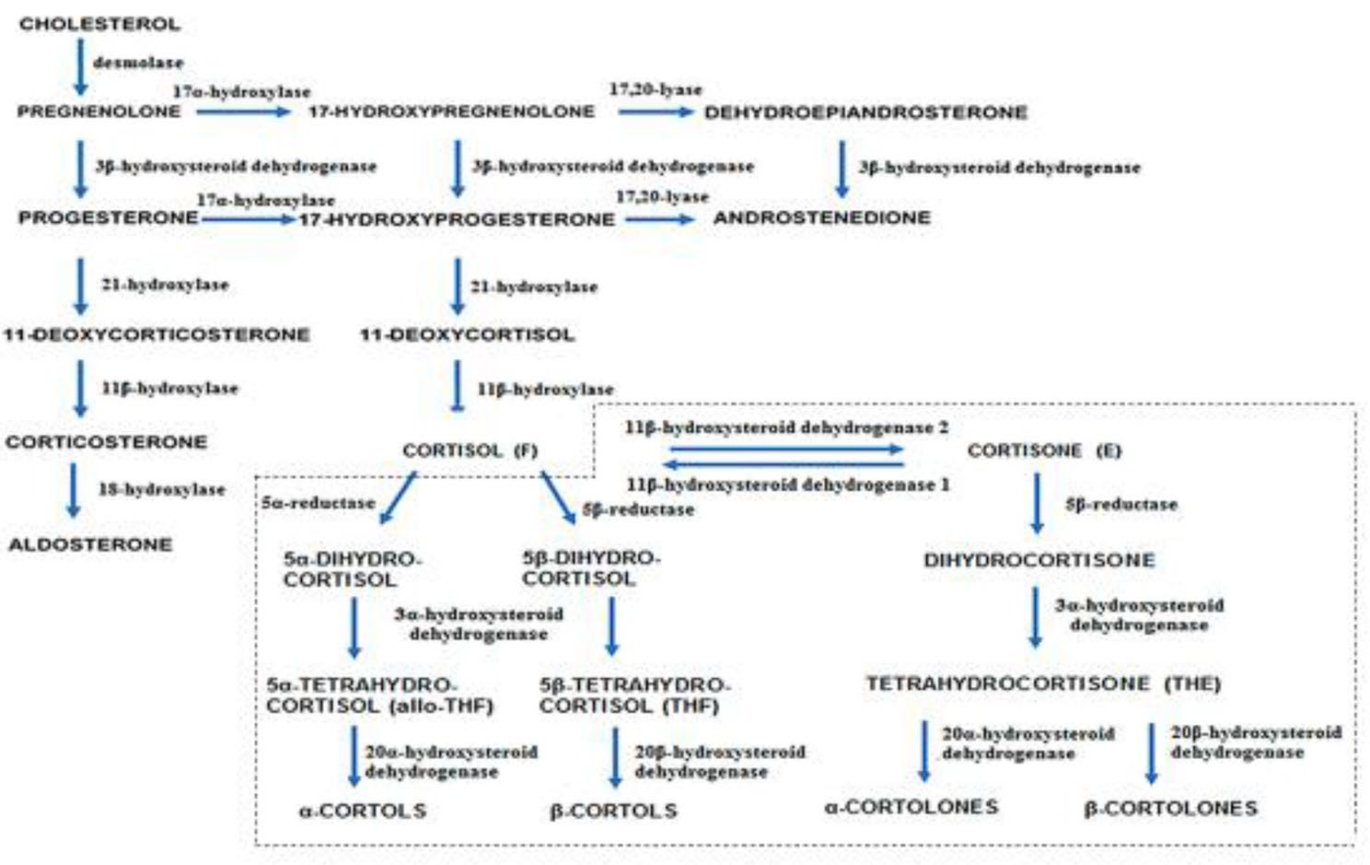
Principal metabolites of cortisol in urine measured by gas chromatography-mass spectrometry. All the metabolites that are in the dotted box are urinary metabolites

**Fig. 3:**
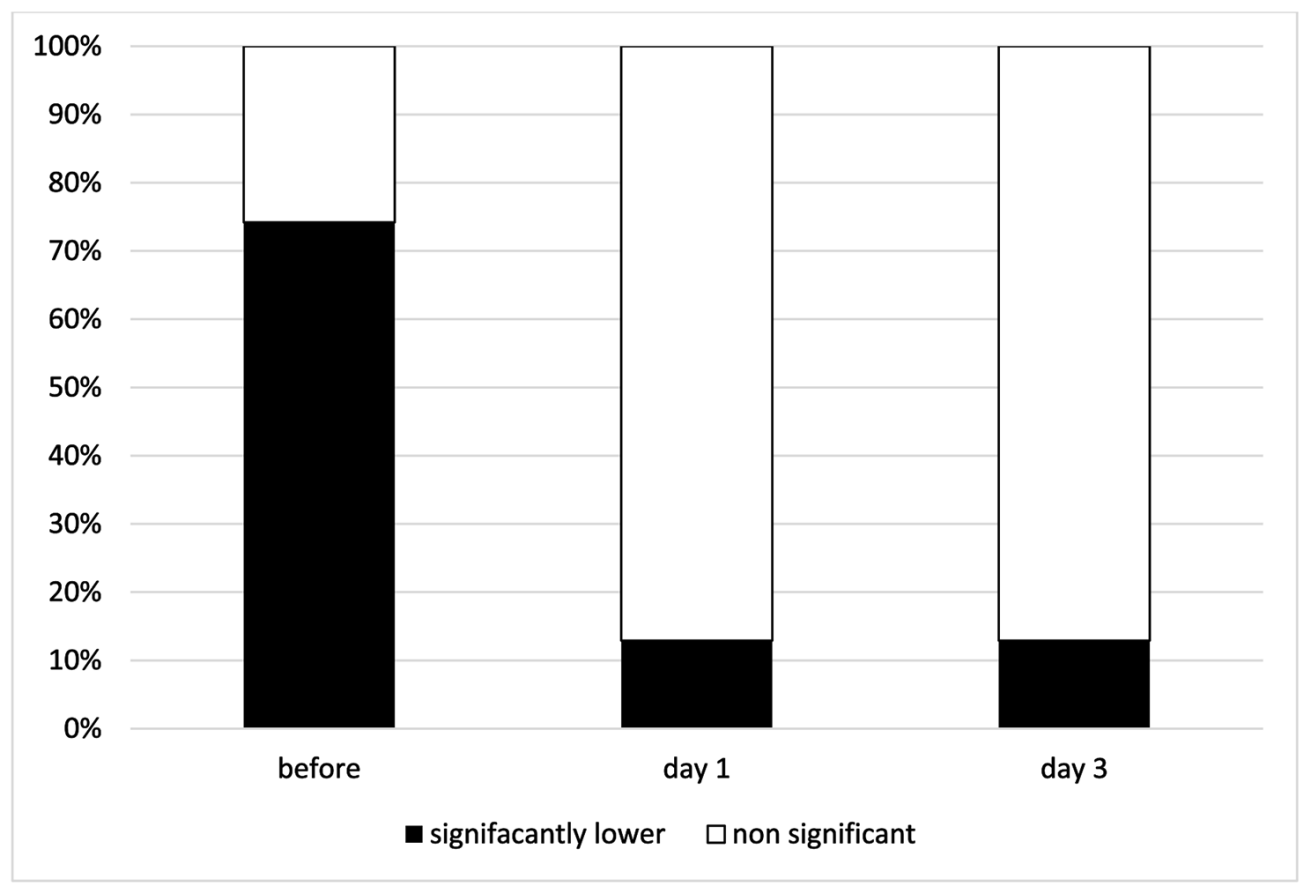
T The relative percentage contribution of urinary steroid metabolites compared between the treatment group and the control group. The black columns represent the percentage of adrenal urinary metabolites that were significantly lower compared to the control group, and the white columns represent the percentage of adrenal urinary metabolites that were comparable to the control group (not significantly different). Before Etanercept treatment more than 70% of the urinary steroid metabolites were significantly lower in the patients as compared to the normal matched controls. One day after Etanercept treatment only 10% of the urinary steroid metabolites remained significantly lower in the patients as compared to the normal matched controls (also comparable to levels on day 3)

None of the metabolites at any time during the collection was significantly higher in the JIA patients than in the control group. The metabolite ratios that reflect enzyme activities before etanercept treatment and 3 days following it are presented in Table 3. The ratio (THE + THF + aTHF)/PT that reflects the activity of CYP21A2 reached a p value of 0.06, and the ratio of ((a-C)+(b-C))/((a-Cl)+(b-Cl)) that reflects the activity of the renal 11β-HSD2 reached a p value of 0.07.

**Table 3:** Urine metabolites ratios that reflect enzyme activities

Enzyme	Ratio	p value
CYP21A2–21-hydroxylase	THE + THF + aTHF)/PT	0.06
CYP17A1 – (17α-hydroxylase/17,20-lyase)	(THE + THF + aTHF)/(THA + TH B + aTHB)	0.39
	(An + Et)/(THA + THB + aTHB)	0.57
CYP11B1 11β-hydroxylase	(THE + THF + aTHF)/100*THS:	0.32
11 β-Hydroxysteroid dehydrogenase type 2 (11β-HSD2)	THE/ (THE + a THF )	0.53
	(a-Cl)+(b-Cl))/(a-C + b-C)	0.38
11 β-Hydroxysteroid dehydrogenase type 1 (11β -HSD1)	(THF + aTHF)/THE:	0.95
	(a-Cl + b-Cl) /(a-Cl + b-Cl):	0.07
5α-reductase/5β reductase	An/Et	0.28
	11-OH-An/11-OH-Et	0.65
	aTHB/THB	0.78
	aTHF/THF	0.89

Significant values (p < 0.05), the p-values were calculated by comparing the metabolite ratios that reflect enzyme activities before etanercept treatment and 3 days following it

## Discussion

JIA is the most common rheumatic disease in children, Several studies have shown that patients with JIA have low early morning serum cortisol levels and low urinary free cortisol levels [12] [13].

Studies in adult patients with RA have shown that anti-TNFα therapy normalized the HPA axis, by increasing ACTH and cortisol levels and by decreasing the ratio of ACTH to cortisol [14]. These findings suggest that anti-TNFα treatment has an effect on the HPA axis and that it improves adrenal hormone secretion, but comparable findings have never been reported in pediatric JIA patients [13] [14] [15] [16].

The aim of the current study was to evaluate the role of anti-TNF α therapy on the pediatric adrenal steroid hormone metabolism as reflected by urinary steroid metabolites determined by GC-MS. We found that 21 urine metabolite levels were significantly lower in JIA patients before they underwent etanercept treatment: 16 of them normalized 1 day post-treatment and only 5 remained lower than normal after 3 days. The five urinary metabolites included two C19 metabolites An and 11-OH-An, two C21 intermediate metabolites Pt and P5D and one cortisol metabolite α-Cl.

Jäättelä et al. [8] showed in a seminal study that TNFα inhibits the expression of mRNAs of adrenal cytochrome P450 oxygenases, CYP11A1 (cholesterol side-chain cleavage enzyme/cholesterol 20-22-desmolase), P450c11 (11 beta-hydroxylase/18-hydroxylase/18-methyl oxidase), P450c17 (17 alpha-hydroxylase/17, 20-lyase) and P450 C21 (21-hydroxylase) in human fetal adrenals. The decrease in gene expression was accompanied by a decrease in cortisol but not in either dehydroepiandrosterone sulphate (DHEAS) or in androstenedione. All of the effects of TNFα were neutralized by the addition of monoclonal anti-TNFα antibody. These results demonstrated that TNFα suppresses the synthesis of cortisol and shifts the steroid secretory pattern towards androgen production, at least partially, by suppressing the accumulation of mRNAs for adrenal cytochrome P450 oxygenases.

The finding that five urine metabolites (An, 11-OH-An, Pt, P5D and a-Cl) remained significantly low 3 days after etanercept treatment may indicate that the enzymes that are involved in their metabolism are more affected by TNFα than the other enzymes. Urine metabolite ratios reflect enzymatic activities. Although there was no significant difference in most of those ratios between the control group and the patient group before and after etanercept treatment, the ratios that reflect 21 hydroxylase and 11β-hydroxysteroid dehydrogenase (11β-HSD) type 2 activity were very close to being significantly different between the groups. That finding indicates that these 2 enzymes were more affected by TNFα in the patient group. Notably, these 2 enzymes are involved in cortisol production and cortisol conversion to cortisone. Active cortisol is converted to inactive cortisone mainly by the kidney via 11β-hydroxysteroid dehydrogenase (11β-HSD) type 2 in order to protect the nonspecific mineralocorticoid receptor from activation by cortisol. The liver is the major organ for converting inactive cortisone to active cortisol, and it does so via 11β-HSD1 (Fig − 1). The expression of 11β-HSD1 is enhanced by TNF and proinflammatory cytokines. mainly at the intracellular compartment, however, it can induce a change in the HPA axis [15].

Although most of the patients in the current study were either free of active disease or had mild disease activity, their urine cortisol metabolites were low compared to the normal controls indicating that they had an interrupted HPA axis and that anti-TNFα therapy had a significant effect restoring the HPA axis in these patents.

One of the challenges in treating JIA patients is to decide when to stop treatment, such as anti-TNF α, and to try to predict who will need to resume therapy. Toward this end, the interrupted HPA axis in JIA patients with active as well as subclinical disease may be able to identify the patients for whom it is safe to stop therapy Furthermore the recovery of the HPA axis by anti TNFα improves not only the inflamed joints but also other important health aspects, such as growth, bone health and general wellbeing.

Our study has a few limitations that bear mention. The main limitation is our small number of patients, and another is their range of ages and different stages of sexual development that may influence the HPA axis. We tried to overcome this latter limitation by matching them with a control group by age and Tanner stage development. Another drawback is that we do not have 24-hour urinary samples to precisely calculate the excretion rates of urinary steroid hormone metabolites and thereby assess hormonal production rates.

Two patients were treated during the study with methotrexate. Methotrexate effect as an anti-inflammatory drug is mainly due to its effect on the accumulation of adenosine triphosphate and its ability to inhibit nucleotide synthesis and cell division. [16]. Given that methotrexate’s is not involved in cortisol metabolism, we did not exclude these patients from the study.

## Conclusions

The findings of the current study demonstrated that anti-TNFα treatment has a rapid effect on urine adrenal metabolites in children with JIA. The therapeutic effect of anti-TNF α treatment in JIA may be related to its effect on the restoration of adrenal function and cortisol levels.

A larger study is warranted to confirm our findings and to evaluate whether the urinary steroid metabolome can be used as a predictor to assess and monitor disease activity and serve as a predictor for disease flare.

- Subheading- Anti-TNFα treatment has a rapid effect on urine adrenal metabolites in children with JIA. The therapeutic effect of anti-TNFα treatment in JIA may be related to its effect on the restoration of adrenal function and cortisol levels. Steroid metabolome is suitable for assessing and monitoring the disease activity in JIA as well as for predicting disease flare.

## Data Availability

The datasets used and/or analysed during the current study are available from the corresponding author on reasonable request.

## Abbreviations of steroids

An: 5α-Androstane-3α-ol-17-one (Androsterone)
Et: 5β-Androstane-3α-ol-17-one (Etiocholanolone)
DHEA: 5-Androstene-3β-ol-17-one (Dehydroepiandrosterone)
16 α-OH-DHEA: 5-Androstene-3β,16α-diol-17-one
A5-3β,17α a: 5-Androstene-3β,17α-diol diol
A5-3β,17β: 5-Androstene-3β,17β-diol (Androstenediol-17β)
A5T- 16α: 5-Androstene-3β,16 β,17βb-triol (Androstenetriol-16α)
PD: 5β-Pregnane-3α,20α-diol (Pregnanediol)
PT: 5β-Pregnane-3α,17α,20α-triol (Pregnanetriol)
P5D: 5-Pregnene-3β,20α-diol (Pregnanediol)
P5T-17α: 5-Pregnene-3β,17α,20α-triol (17α-Pregnenetriol)
Po-5β,3α: 5β-Pregnane-3α,17αdiol-20-one (17α-OH-Pregnanolone)
Po-5α,3α: 5α-Pregnane-3α,17α-diol-20-one
F: 4-Pregnene-11β,17α,21-triol-3,20-dione (Cortisol)
THE: 5β-Pregnane-3α,17α,21-triol-11,20-dione (TH-Cortisone)
THF: 5β-Pregnane-3α,11β,17α,21-tetrol-20-one (TH-Cortisol)
α-THF: 5α-Pregnane-3α,11β,17α,21-tetrol-20-one (Allo-TH-Cortisol)
α-Cl: 5β-Pregnane-3α,17α,20αa,21-tetrol-11-one (α-Cortolone)
β-Cl: 5β-Pregnane-3α,17α,20β,21-tetrol-11-one (β-Cortolone)
α-C: 5β-Pregnane-3α,11β,17α,20α,21-pentol (α -Cortol)
β-C: 5β-Pregnane-3α11β,17α,20β,21-pentol (β -Cortol)
6β -OH-F: 4-Pregnene-6β,11β,17α,21-tetrol-3,20-dione
20α-DHF: 4-Pregnene-11β,17α,20α,21-tetrol-3-one
11-OH-An: 5α-Androstane-3α,11β-diol-17-one
11-O-An: 5α-androstane-3α-ol-11,17-dione
11-OH-Et: 5βb-androstane-3α,11β-diol-17-one
11-O-PT: 5β-Pregnane-3α,17α,20α-triol-11-one
THA: 5β-Pregnane-3α,21-diol-11,20-dione
THB: 5β-Pregnane-3α,11β,21-triol-20-one (TH-Corticosterone)
α-THB: 5α-Pregnane-3α,11β,21-triol-20-one (Allo-TH-Corticosterone)
THS: 5β-Pregnane-3α,17α,21-triol-20-one
E1: Estrone
E2: Estradiol
E3: Estriol
T: Testosterone
